# Genome sequence of *Desulfitobacterium hafniense *DCB-2, a Gram-positive anaerobe capable of dehalogenation and metal reduction

**DOI:** 10.1186/1471-2180-12-21

**Published:** 2012-02-08

**Authors:** Sang-Hoon Kim, Christina Harzman, John K Davis, Rachel Hutcheson, Joan B Broderick, Terence L Marsh, James M Tiedje

**Affiliations:** 1 Center for Microbial Ecology, Michigan State University, East Lansing, MI, USA; 2 Department of Biology, Columbus State University, Columbus, GA, USA; 3 Department of Chemistry & Biochemistry, Montana State University, Bozeman, MT, USA; 4 Department of Microbiology and Molecular Genetics, Michigan State University, East Lansing, MI, USA

## Abstract

**Background:**

The genome of the Gram-positive, metal-reducing, dehalorespiring *Desulfitobacterium hafniense *DCB-2 was sequenced in order to gain insights into its metabolic capacities, adaptive physiology, and regulatory machineries, and to compare with that of *Desulfitobacterium hafniense *Y51, the phylogenetically closest strain among the species with a sequenced genome.

**Results:**

The genome of *Desulfitobacterium hafniense *DCB-2 is composed of a 5,279,134-bp circular chromosome with 5,042 predicted genes. Genome content and parallel physiological studies support the cell's ability to fix N_2 _and CO_2_, form spores and biofilms, reduce metals, and use a variety of electron acceptors in respiration, including halogenated organic compounds. The genome contained seven reductive dehalogenase genes and four nitrogenase gene homologs but lacked the Nar respiratory nitrate reductase system. The *D. hafniense *DCB-2 genome contained genes for 43 RNA polymerase sigma factors including 27 sigma-24 subunits, 59 two-component signal transduction systems, and about 730 transporter proteins. In addition, it contained genes for 53 molybdopterin-binding oxidoreductases, 19 flavoprotein paralogs of the fumarate reductase, and many other FAD/FMN-binding oxidoreductases, proving the cell's versatility in both adaptive and reductive capacities. Together with the ability to form spores, the presence of the CO_2_-fixing Wood-Ljungdahl pathway and the genes associated with oxygen tolerance add flexibility to the cell's options for survival under stress.

**Conclusions:**

*D. hafniense *DCB-2's genome contains genes consistent with its abilities for dehalogenation, metal reduction, N_2 _and CO_2 _fixation, anaerobic respiration, oxygen tolerance, spore formation, and biofilm formation which make this organism a potential candidate for bioremediation at contaminated sites.

## Background

Species of *Desulfitobacterium *are Gram-positive, strictly anaerobic bacteria that belong to the *Firmicutes*, *Clostridia*, *Clostridiales *and *Peptococcaceae*. The genus is currently composed of six described species, *D. metallireducens, D. dichloroeliminans, D. dehalogenans, D. chlororespirans, D. aromaticivorans*, and *D. hafniense *[1,2]. Most of *Desulfitobacterium *species were isolated for their ability to reductively dehalogenate organic compounds which are, in some cases, highly resistant to aerobic biodegradation and toxic to bacteria [1]. Dehalorespiration, in which energy is acquired under anaerobic conditions by coupling of the reduction of halogenated organic compounds to the oxidation of electron donors, has been intensively studied in *Desulfitobacterium *and *Dehalococcoides *as potential bioremediation agents at contaminated sites [1,3]. *Desulfitobacterium *is distinguished in its use of a broad range of electron acceptors (As(V), Fe(III), U (VI), Cr(VI), Se(VI), Mn(IV), S°, SO_3_^-2^, S_2_O_3_^-2^, NO_3_^-^, CO_2_, fumarate, DMSO, and AQDS [1]) as well as electron donors (H_2_, formate, L-lactate, butyrate, butanol, crotonate, malate, pyruvate, and ethanol). *D. aromaticivorans*, a recently discovered iron reducer, can use aromatic hydrocarbons including toluene, phenol, *p*-cresol, and *o*-xylene as carbon and energy sources [2].

*Desulfitobacterium hafniense *DCB-2 was first isolated from a municipal sludge in Denmark based on its ability to dechlorinate halogenated phenols [4]. Its ability to use metal ions as electron acceptors was reported for Fe(III), Mn(IV), Se(VI), and As(V) [5,6]. The strain also uses non-metal electron acceptors such as S°, SO_3_^-2^, S_2_O_3_^-2^, NO_3_^-^, fumarate, isethionate, DMSO, 2,4,6-trichlorophenol, and other chlorinated phenols [4,6,7]. Nine strains have been identified to date that belong to *D. hafniense *species including *D. hafniense *Y51 which was isolated from a Japanese soil contaminated with tetrachloroethene [8], and for which the complete genome sequence was reported [1,9]. Although *D. hafniense *strains DCB-2 and Y51 are very closely related (> 99% identity in 16S rRNA sequence) and share many common metabolic features, important differences exist in certain aspects of metabolism such as the presence of a respiratory nitrate reduction system in Y51, the potential substrate use of 4-hydroxy-2-oxovalerate by DCB-2, and the different dehalogenation capacities. DCB-2 contains seven reductive dehalogenase (RDase) genes, mostly responsible for the dechlorination of various chlorophenols, whereas Y51 contains two RDase genes and is capable of dechlorinating tetrachloroethene (PCE) to *cis*-1,2-dichloroethene [8,10]. We report here on the genome sequence of *D. hafniense *DCB-2 with specific reference to its metal reduction and dehalogenation abilities, in addition to the comparison with strain Y51. We also provide results from expression arrays that complement the genomic data.

## Results and discussion

### Differences in *D. hafniense *DCB-2 and Y51 genomes

**Figure 1:**
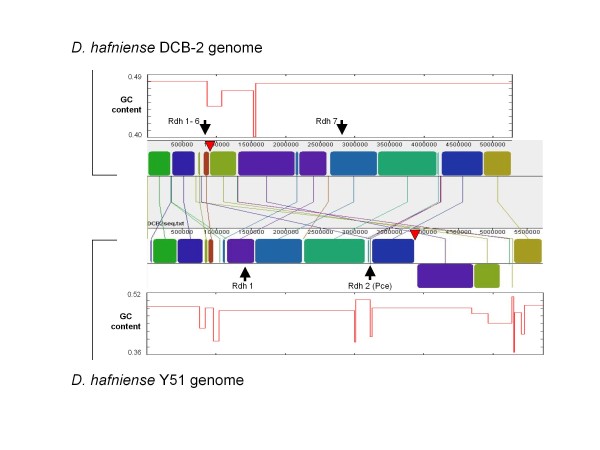
**Alignment and GC-profiles of the genomes of *D. hafniense *DCB-2 and *D. hafniense *Y51**. Alignment of the two genomes, shown with colored blocks of DNA and connecting lines, was performed by using Mauve v 2.3.1 with a view of 24 LCBs (locally collinear blocks). The lines between the genomes indicate the homologous regions in each genome. Translocation of a 1.22 mb DNA segment is seen as two contiguous blocks colored purple and green. Two transposase genes found next to the 1.22 mb DNA segment are indicated as red triangles. Positions of reductive dehalogenase (Rdh) operons in each genome are indicated. The two outer panels show the corresponding GC profiles of the two genomes, depicted as compositionally distinct domains. The profiles were obtained by using GC-Profile program which was developed based on a segmentation algorithm and cumulative GC profile technique.

*D. hafniense *DCB-2 carries a single circular genome of 5,279,134 bp with a total of 5,042 predicted genes (Table 1) excluding 70 pseudogenes and gene remnants. Five rRNA operons and 74 tRNA genes constitute a total of 89 RNA genes leaving 4,953 protein-encoding genes (CDS). *D. hafniense *Y51 contains six rRNA operons and 59 tRNA genes, and has a slightly larger genome by 448 kb (8.5% of the DCB-2 genome) with 166 more genes [9]. Similar proportions of genes were observed for transmembrane proteins and for twin-arginine signal peptide proteins (Table 1). However, genes for signal peptide proteins were found more abundantly in the genome of DCB-2 (725 genes) than Y51 (661 genes). The number of horizontally transferred genes that putatively originated from organisms above the level of the *Peptococcaceae *family was 264 in DCB-2 and 285 in Y51. When the two genomes were compared at the level of CDS, the number of genes found only in the DCB-2 genome was 614. Among them, 341 were with no functional hit. The Y51 genome had 583 unique genes including 319 with no functional hit. The larger number of the unique genes in DCB-2, despite its smaller number of total CDS, suggests that the Y51 genome contains more gene duplications, as indicated by the number of paralogs in Table 1. Among the DCB-2 genes with no homolog in Y51, most notable are the genes for reductive dehalogenases (RDases) and prophage-like sequences. Six out of the seven RDase genes in DCB-2 are located in a cluster, while there are only two in Y51 (Figure 1) [9]. Multiple prophage sequences that are unique to each genome were found in both strains. The DCB-2 genome contains at least three prophage-like sequences though none of them contained a full gene set in comparison with the known prophage equivalents. A fourteen-gene-encoding prophage sequence spanning 11.8-kb (Dhaf_1454-1467) appears to belong to the phage HK97 family, a lambda-like double-stranded DNA bacteriophage. The genome of the functional *Escherichia coli *phage HK97 contains 74 genes on a 39.7-kb genome [11]. Also found only in *D. hafniense *DCB-2 were genes for rhamnan biosynthesis (Dhaf_4461-4467) and 4-hydroxy-2-oxovalerate aldolase (Dhaf_1245) which converts 4-hydroxy-2-oxovalerate to acetaldehyde and pyruvate. A *nar *operon was identified in the Y51 genome that is responsible for respiratory nitrate reduction which was absent in DCB-2.

**Table 1:** Genome features of *D.hafniense *DCB-2 and *D. hafniense *Y51

Genome Features	*D. hafniense *DCB-2	*D. hafniense *Y51
Bases	5279134	5727534

GC (%)	0.48	0.47

Genes	5042	5208

CDS	4953	5060

rRNA; 5S, 16S, 23S	5, 5, 5	6, 6, 6

tRNA	74	59

Orthologs	4834 (95.9%)	4834 (92.8%)

Paralogs	1245 (24.7%)	1369 (26.3%)

Signal P*	725 (14.4%)	661 (12.7%)

Transmembrane P**	934 (18.5%)	976 (18.7%)

Tat signal P***	414 (8.2%)	442 (8.5%)

**Horizontally transferred**	264	285

**Genes with no homolog in other genome:**		

total	614	583

in COG	164	186

no functional hit	341	319

notable genes	reductive dehalogenase	Nar nitrate reductase

*Data obtained using SignalP 3.0

**Data obtained using TMHMM Server v. 2.0

***Potential Tat proteins with no Tat motif are also included. Data obtained using TatP 1.0

The genome of *D. hafniense *Y51 was reported to have the most uneven lengths of chromosome arms which result from the bidirectional replication of a circular chromosome at the replication origin. Based on its GC skew plot [(G-C)/(G+C)], the Y51 genome is predicted to have the lagging strand (negative GC-skew value) roughly twice as long as the leading strand (positive GC-skew value) [9]. In contrast, the DCB-2 genome had a slightly longer leading strand (the ratio of 1.3:1). Alignment of the two genomes revealed that a translocation of a 1.22 Mb DNA segment accounted for the GC skew difference (Figure 1). The immediate junctions of this segment were identified by an IS116/IS110/IS902 family transposase gene (Dhaf_0814) in DCB-2 and an IS4 family transposase gene (DSY3435) in Y51 (Figure 1), strongly implicating these insertion sequences in the translocation. The GC content profiles obtained by a segmentation algorithm show that the *D. hafniense *Y51 genome contains broader regions of unusually low GC content, which appear to be occupied by prophage genomes and horizontally transferred sequences of unknown origin (Figure 1).

### Carbon metabolism

The *D. hafniense *DCB-2 genome encodes genes for functional glycolysis, gluconeogenesis, and pentose phosphate pathways. The genome lacks the alternate Entner-Doudoroff pathway for glucose breakdown, which is used by many Gram-negative aerobic bacteria and Archaea [12]. Genes associated with sugar phosphotransferase system (PTS) were not found, consistent with the cell's inability to utilize sugar substrates for growth [4]. Tryptophan is the only known substrate other than pyruvate that is used for fermentative cell growth in this organism [5]. Two copies of the gene (Dhaf_1324 and Dhaf_2460) coding for tryptophanase which converts tryptophan to indole, pyruvate, and ammonia were identified in association with two permease genes (Dhaf_1325 and Dhaf_2459). These gene sets were also observed in Y51 (DSY4041-4042 and DSY1331-1332).

Complete biosynthetic pathways are present for the formation of amino acids, nucleic acid precursors, as well as fatty acids and phospholipids. The genome also encodes complete biosynthetic pathways for various enzyme cofactors and prosthetic groups including NAD(P), menaquinone, heme, thiamine pyrophosphate, pyridoxal phosphate, riboflavin, pantothenate, folate, and biotin. However, the genome of *D. hafniense *DCB-2 appears to lack a gene for dihydrofolate reductase, a ubiquitous enzyme that is required for the synthesis of tetrahydrofolate (THF). THF is involved in one-carbon transfer reactions and in the synthesis of purine bases, glycine, and serine. The gene was neither found in the Y51 genome, nor in those of other members of the *Peptococcaceae *family listed in IMG (Integrated Microbial Genomes), suggesting that this group of organisms may have evolved an unconventional dihydrofolate reductase for the synthesis of THF.

**Figure 2:**
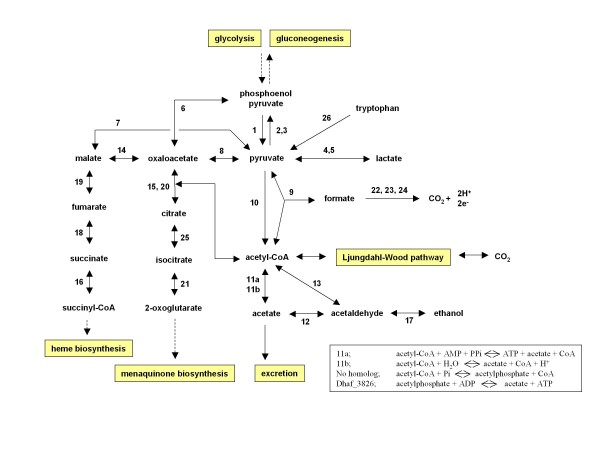
**Carbon metabolic pathways of *D. hafniense *DCB-2**. The pathways were constructed based on the presence or absence of key metabolic genes in *D. hafniense *DCB-2. The acetyl-CoA degradation and related genes are shown in more detail (boxed). Enzymes for the numbered reactions in figure are listed below with their potential genes; **1**. pyruvate kinase; Dhaf_2755. **2**. phosphoenolpyruvate synthase; Dhaf_1117, Dhaf_1622, Dhaf_3294. **3**. pyruvate, phosphate dikinase; Dhaf_1046, Dhaf_4240, Dhaf_4251. **4**. D-lactate dehydrogenase (cytochrome); Dhaf_3228, Dhaf_4382. **5**. L-lactate dehydrogenase; Dhaf_1965. **6**. PEP carboxykinase; Dhaf_1134. **7**. malate dehydrogenase (NADP^+^); Dhaf_0902, Dhaf_3085. **8**. pyruvate carboxyltransferase; Dhaf_1012, Dhaf_1059. **9**. pyruvate formate-lyase; Dhaf_0366, Dhaf_1246, Dhaf_4905. **10**. pyruvate flavodoxin/ferredoxin oxidoreductase; Dhaf_0054, Dhaf_4766. **11a**. acetate-CoA ligase; Dhaf_0467. **11b**. acetyl-CoA hydrolase/transferase; Dhaf_0603, Dhaf_2858, Dhaf_4529. **12**. aldehyde dehydrogenase (NAD^+^); Dhaf_2181. **13**. acetaldehyde dehydrogenase (acetylating); Dhaf_2180. **14**. malate dehydrogenase; Dhaf_1799, Dhaf_4412. **15**. citrate lyase; Dhaf_4206. **16**. succinate-CoA ligase (ADP-forming); Dhaf_0192, Dhaf_2066. **17**. alcohol dehydrogenase; Dhaf_2180, Dhaf_0588. **18**. succinate dehydrogenase; Dhaf_0743-0745. **19**. fumarase; Dhaf_4397. **20**. citrate synthase; Dhaf_0903. **21**. isocitrate dehydrogenase (NADP^+^); Dhaf_1523. **22**. hydrogen:quinone oxidoreductase; Dhaf_2742. **23**. hydrogenase (ferredoxin); Dhaf_0805, Dhaf_3270, Dhaf_3368. **24**. formate dehydrogenase; Dhaf_1398, Dhaf_1509, Dhaf_4271. **25**. aconitase; Dhaf_1133. **26**. tryptophanase; Dhaf_1324, Dhaf_2460.

The tricarboxylic acid cycle (TCA) of *D. hafniense *DCB-2 and Y51 appears incomplete since they lack the gene coding for 2-oxoglutarate dehydrogenase, and the cycle lacks the anaplerotic glyoxylate bypass (Figure 2). In most autotrophic bacteria and anaerobic Archaea, the TCA cycle operates in a reductive, biosynthetic direction [13]. In line with this observation, DCB-2 and Y51 are apparently capable of performing the reductive TCA cycle due to the possession of additional enzymes such as fumarate reductase and citrate lyase to potentially bypass the unidirectional steps of the conventional oxidative TCA cycle [14] (Figure 2). However, the reconstruction of the TCA cycle based solely on genome sequence should be carefully addressed, as observed in *Clostridium acetobutylicum *where both functional oxidative and reductive TCA cycles were confirmed experimentally in contrast to the previous genomic interpretation [15].

*D. hafniense *DCB-2 appears to use two-carbon substrates selectively for the synthesis of acetyl-CoA or for its degradation to acquire ATP. For example, ethanol, but not acetate, was shown to support cell growth when an electron acceptor, As(V), was provided [6]. While both DCB-2 and Y51 contain acetate kinase (Dhaf_3826), they lack the gene for phosphate acetyltransferase, making the cells unable to gain ATP from acetyl-CoA degradation. However, they contain an alternative acetate-CoA ligase (Dhaf_0467 and DSY0515) that could be used to gain ATP from AMP by directly converting acetyl-CoA to acetate (boxed in Figure 2). The presence of multiple copies of acetaldehyde dehydrogenase genes in both strains (Dhaf_0356, 1244, 4892, 4906, and DSY0244, 0406, 4993, 5007) suggests that acetaldehyde is an important intermediate in two-carbon metabolism.

#### Wood-Ljungdahl pathway

**Figure 3:**
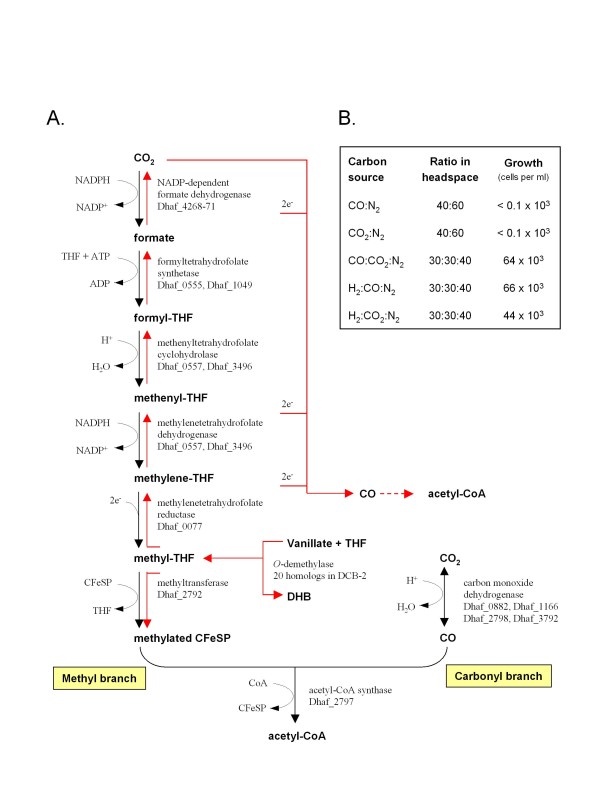
**The Wood-Ljungdahl pathway and CO_2 _fixation in *D. hafniense *DCB-2**. (a) Key enzymes involved in the Wood-Ljungdahl pathway and the corresponding gene homologs are indicated. The pathway depicts the methyl branch (left) and the carbonyl branch (right) prior to forming acetyl-CoA. Reactions for the methyl group that is derived from vanillate demethylation are indicated with red arrows; DHB, 3,4-dihydroxybenzoate. Homolog searches were performed by BLASTP with cutoff values of 1e-2 (E-value) and 30% identity in amino acid sequence. (b) Autotrophic cell growth of *D. hafniense *DCB-2 as measured by total number of the cell per ml culture.

The *D. hafniense *DCB-2 genome contains a complete gene set for the Wood-Ljungdahl (or reductive acetyl-CoA) pathway. Figure 3 shows the key enzymes and corresponding genes in the pathway of CO_2 _fixation, where two CO_2 _molecules are reduced to a methyl- and a carbonyl-group, and are ligated with CoA to form acetyl-CoA. Protein sequences and organization of the genes in the pathway are highly similar to those of *Moorella thermoacetica*, the model acetogenic bacterium extensively studied for the elucidation of this pathway [16]. While genes encoding enzymes that convert CO_2 _to formate and then to methyl-tetrahydrofolate (Figure 3a, methyl branch) are found scattered around the *D. hafniense *DCB-2 genome, genes encoding enzymes that constitute the CO dehydrogenase/acetyl-CoA synthase (CODH/ACS) and other related enzymes are localized in an eight-gene operon, Dhaf_2792-2799 (Figure 3a, carbonyl branch). The methyl branch of DCB-2 appears to be bidirectional (CO_2_-forming as well as methyl-forming) and used for the growth on phenyl methyl ethers such as lignin-derived vanillate as electron donors (Figure 3) [17,18]. Fumarate or 3-chloro-4-hydroxyphenylacetate was required as an electron acceptor for the growth on vanillate [17]. As indicated in Figure 3a, the methyl group of vanillate cleaved by *O*-demethylase enters the methyl branch to form CO_2 _while generating reducing power that could be used to convert CO_2 _to CO. Twenty homologs were identified in the DCB-2 genome for the gene encoding a vanillate-specific *O*-demethylase corrinoid protein (*odmA*) while 15 were found in Y51 [9,19].

*M. thermoacetica *grows autotrophically on CO_2 _and H_2 _using the Wood-Ljungdahl pathway, but since no ATP is gained from substrate-level phosphorylation by this pathway, anaerobic respiration is implicated [16]. Establishment of a proton gradient through formate hydrogenlyase activity was postulated as one of potential mechanisms for energy generation [16]. Since DCB-2 has genes for the same pathway for CO_2 _fixation and for formate hydrogenlyase (Dhaf_4269-4271), we tested its ability to grow solely on CO_2 _and H_2_. While DCB-2 grew under this condition compared to a no-H_2 _control (Figure 3b), the growth was not as robust as *M. thermoacetica *run in parallel. In addition, the growth results also indicate that CO was metabolized, presumably oxidized to form H^+ ^and CO_2 _by CO dehydrogenase encoded by four gene copies (Figure 3a). The CO_2 _would then enter the methyl branch of the Wood-Ljungdahl pathway to produce a methyl group. In the photosynthetic bacterium *Rhodospirillum rubrum*, CO induces CO dehydrogenase (CooS) and CO-tolerant hydrogenase (CooF), which allows cell growth in a CO-dependent manner in the dark [20]. By BLAST search we identified a gene similar to *cooF *(E value of 2e-49) located within a twelve-gene operon (Dhaf-4277-4288). The operon also encodes gene homologs for *E. coli *hydrogenases 3 and 4, both of which are part of formate hydrogenlyase complexes [21]. Similar to NADH dehydrogenase and to the CooF of *R. rubrum, E. coli *hydrogenase 4 has been implicated in proton translocation [21]. Other genes in the operon include two sporulation-related genes, *ygfCD*, and genes for phosphate starvation-inducible protein PhoH, a phosphohydrolase, and a diacylglycerol kinase.

### Energy metabolism

#### Electron transport chain

Ubiquinone and menaquinone in bacteria are lipid-soluble molecules that shuttle electrons between the membrane proteins in the electron-transport chain. In *Escherichia coli*, ubiquinone is used for aerobic and nitrate respiration, while menaquinones are used for fumarate, trimethylamine oxide (TMAO), and dimethyl sulfoxide (DMSO) (anaerobic) respiration [22]. Many Gram-positive aerobes contain only menaquinones [23]. *Bacillus subtilis *which can grow both aerobically and anaerobically uses menaquinone for aerobic, nitrate, and nitrite respiration [24]. The *D. hafniense *DCB-2 genome lacks the ubiquinone biosynthesis pathway but contains a complete menaquinone biosynthesis pathway, enabled by a hexacistronic operon (*menBCDEFH*; Dhaf_0469-0474) and two separately located genes, *menA *(Dhaf_4028) and *menG *(Dhaf_3067).

Transfer of electrons to a quinone pool is largely mediated by a respiratory-chain enzyme NADH:quinone oxidoreductase. The enzyme complex of DCB-2 is encoded by an 11 gene operon (Dhaf_3741-3751). Besides NADH, formate serves as an important electron donor to a menaquinone pool in anaerobic respiration with substrates such as nitrate, DMSO, and TMAO. Oxidation of formate to CO_2_, 2H^+^, and 2e^- ^is catalyzed by quinone-dependent formate dehydrogense (FDHase) while NAD-dependent FDHase directs carbon fixation by converting CO_2 _to formate which is subsequently used in the Wood-Ljungdahl pathway. Two putative FDHase operons were identified in *D. hafniense *DCB-2 (*fdh*-1 and *fdh*-2). The quinone-dependent FDHase operon, *fdh*-1 (Dhaf_4269-4271), contains a complete set of three genes encoding a catalytic molybdopterin enzyme FdhA, a 4Fe-4S protein FdhB, and a quinone-binding cytochrome FdhC. Our transcriptomic study indicated that this operon was inducible when ferric ion was used as the electron acceptor for respiration [25], suggesting that the quinone-dependent FDHase may play a role in dissimilatory ferric ion reduction. Genes encoded in *fdh*-2 (Dhaf_1396-1398) are consistent with its role as NAD-dependent FDHase, with genes encoding a selenocysteine-containing catalytic subunit FdhA, and two other subunits, FdhB and FdhC, both having NADH dehydogenase activity. A fourth gene was identified within the operon, putatively encoding methenyl-THF (tetrahydrofolate) synthetase. This enzyme catalyzes the interchange of 5-formyl-THF to 5-10-methenyl-THF in the Wood-Ljungdahl pathway.

#### Cytochromes and oxidoreductases

Dissimilar to other metal reducers, *D. hafniense *DCB-2 contains a small number of genes for *c*-type cytochromes with only ten such genes, in comparison with 103 in *Geobacter sulfurreducens *and 91 in *G. metallireducens, where c*-type cytochromes are implicated in Fe(III) and U(VI) reduction [26,27]. Eight annotated *c*-type cytochrome genes in *D. hafniense *DCB-2 are associated with the reductions of nitrite (Dhaf_3630, Dhaf_4235), sulfite (Dhaf_0258), fumarate (Dhaf_3768, Dhaf_4309), and TMAO (Dhaf_1279, Dhaf_4696, Dhaf_4918), but the two others have no implicated function. They are Dhaf_3639 encoding a diheme-containing cytochrome with no linked gene and Dhaf_3269 linked with two NiFe hydrogenase subunit genes forming a unique gene organization among all sequenced genomes in IMG other than the Y51 genome. Genes for cytochrome *bd *quinol oxidase, CydAB, which catalyzes quinol-dependent oxygen uptake, were identified in the DCB-2 genome (Dhaf_1310-1311). This enzyme has been reported to play an important role in microaerobic nitrogen fixation in *Klebsiella pneumoniae*, since a mutation in this gene severely hampered that cell's ability to fix nitrogen [28].

**Figure 4:**
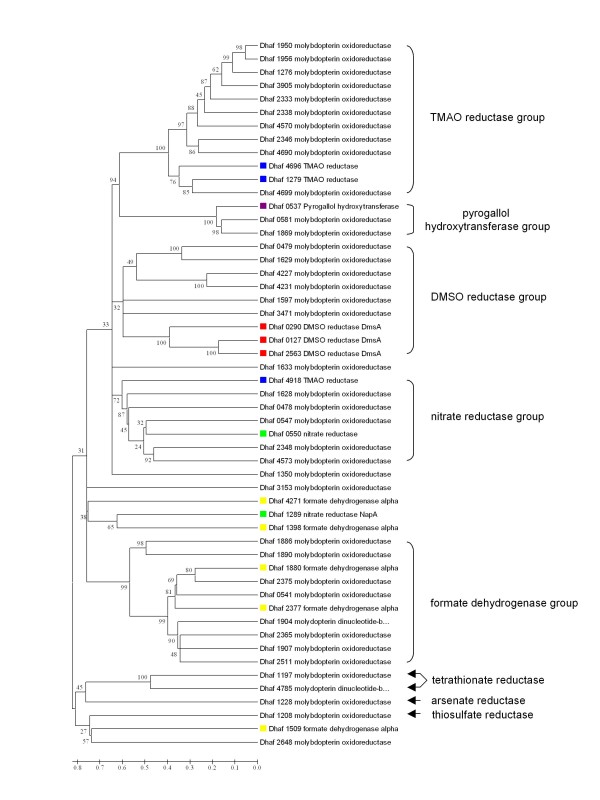
**Phylogenetic tree derived from 53 molybdenum-binding oxidoreductases**. The tree was constructed by using MEGA 4.1 neighbor-joining method with 500 bootstrap replicates. Genes annotated by IMG are color-coded; blue for TMAO reductase, purple for pyrogallol hydroxytransferase, red for DMSO reductase, green for nitrate reductase, and yellow for formate dehydrogenase. Genes that were newly assigned in this study for their potential protein function are indicated with arrows. Bootstrap values are shown for each node, and the scale indicates the number of amino acid substitutions per site.

Of completed genomes thus far, *D. hafniense *DCB-2 and Y51 have the largest number of molybdopterin oxidoreductase genes (pfam01568), with 53 and 57 genes, respectively. Next in rank are *Eggerthella lenta *DSM 2243 (34 genes), and *Slackia heliotrinireducens *DSM 20476 (25 genes). Members of the molybdopterin oxidoreductase family include formate dehydrogenase, nitrate reductase, DMSO reductase, TMAO reductase, pyrogallol hydroxytransferase, and arsenate reductase. A phylogenetic tree with the 53 molybdopterin sequences reveals seven relatively well-defined groups (Figure 4). BLAST analysis of two outliers reveals that Dhaf_4785 and Dhaf_1197 both code for tetrathionate reductase subunit A of the TtrABC complex that catalyzes reduction of tetrathionate to thiosulfate [29]:

$${\text{S}}_{4}{{\text{O}}_{6}}^{2-}+{\text{H}}_{2}\Leftrightarrow 2{\text{S}}_{2}{{\text{O}}_{3}}^{2-}+2{\text{H}}^{+}$$

Equivalent genes for the 4Fe-4S protein TtrB and the integral membrane protein TtrC were identified as linked genes (Dhaf_4783-4784, Dhaf1195-1196). Another outlier, Dhaf_1208, was found to encode a protein similar (E value of 2e-47) in sequence to thiosulfate reductase subunit A, PhsA, of *Wolinella succinogenes *DSM 1740 [30]. Thiosulfate reductase (PhsABC) of *Salmonella typhimurium *catalyzes dissimilatory anaerobic reduction of thiosulfate to hydrogen sulfide [31]. We observed that thiosulfate in the presence of pyruvate supported a faster growth of *D. hafniense *DCB-2 than pyruvate alone. In the DCB-2 genome, the putative *phsABC *operon contains an additional gene encoding a cytoplasmic chaperone protein (Dhaf_1206-1209). The operon is likely responsible for the observed cell growth on thiosulfate and the reduction of thiosulfate to sulfide in the presence of pyruvate [5]. In addition to the molybdopterin-dependent enzymes that carry out the reductive cleavage of sulfur-sulfur bonds, a molydbdopterin enzyme for the arsenate reduction was also identified (Figure 4. Dhaf_1228). The diversification of molybdoprotein oxidoreductases in *D. hafniense *DCB-2 may provide extensive options for anaerobic energy metabolism.

#### Inorganic electron acceptors

Due to their poor solubility in water, metal-oxides and -hydroxides [such as Fe(III), Mn(III)/(IV)] are challenging substrates for bacterial respiration. Multiheme *c*-type cytochromes were shown to mediate dissimilatory reduction of Fe(III) and Mn(III)/(IV) in the Gram-negative bacteria *S. oneidensis *MR-1- and *G. sulfurreducens *[32-34]. The Gram-positive *D. hafniense *DCB-2 contains no homolog for the multiheme cytochromes but is capable of reducing Fe(III) for energy generation [5,25]. Only three genes potentially encoding *c*-type cytochromes that are not part of known enzyme systems were identified and none of them had a multiheme motif. Total genome transcriptomic studies have generated a few potential candidates for a dissimilatory Fe(III) reductase. Among them, an operon encoding a molybdopterin oxidoreductase gene (Dhaf_1509) is of particular interest since we found a very high level of expression (~40 fold) specifically induced when Fe(III) was the terminal electron acceptor. The operon appears to contain six genes including two rhodanese-family genes, a 4Fe-4S binding domain gene, a polysulphide reductase gene, and a TorD- like chaperone gene (Dhaf_1508-1513). In addition, a decacistronic operon (Dhaf_3547-3556) encoding type IV pilus biosynthesis genes was induced 2-3 fold. In *Geobacter sulfurreducens*, type IV pilus has been implicated in mediating electron transfer from the cell surface to insoluble Fe(III) [35]. A mutant defective in the pilin subunit gene (*pilA*) could not reduce insoluble ferric oxide but was still able to reduce soluble ferric citrate [35]. In our microarray studies, ferric citrate [Fe(III)] and uranyl acetate [U(VI)] induced the type IV pilus biosynthesis operon, but sodium selenate [Se(VI)] did not [25].

Uranium in nuclear waste poses an ecological and human health hazard. Microbial reduction of soluble U(VI) to U(IV) which precipitates as uraninite, has been proposed as a method for the immobilization of uranium *in situ *[36]. *Desulfovibrio desulfuricans *G20 and *Desulfovibrio vulgaris *have been shown to directly reduce U(VI), without the involvement of a respiratory electron transfer [37-39]. Similar to the case of Fe(III) reduction, multiheme *c*-type cytochromes have been postulated in association with U(VI) reduction [38,39]. As an additional mechanism to explain the reduction of cytoplasmic U(VI) in *D. desulfuricans *G20, thioredoxin was proposed to be responsible [40]. *D. hafniense *DCB-2 could reduce U(VI) to U(IV) when pyruvate was provided [25]. Under these conditions, cell growth was significantly inhibited, and long, undivided cells were formed, suggesting that U(VI)/U(IV) is deleterious to cell division. Lactate also supported the cell's growth on U(VI) but it took much longer (a few months) before the growth reached a detectable level [25]. Among ten thioredoxin genes identified in the DCB-2 genome, we found none were induced under U(VI)-reducing conditions. However, a significant induction (4-5 fold) was found for a tricistronic operon, Dhaf_0248-0250, which encodes a putative cytochrome *b*-containing nitrate reductase gamma subunit, a cysteine-rich ferredoxin protein, and a NADH oxydase-like protein. This operon, together with the type IV pilus biosynthesis operon (~10 fold induction), may play roles in the formation and transport of electrons for U(VI) reduction.

Although toxic at higher concentrations (MIC of ~0.1 mM for *Escherichia coli *[41]), selenite is required by microbes as the source for selenocysteine and selenomethionine [42]. Selenocysteine supplies selenium to glycine reductase, formate dehydrogenase, and NiFeSe hydrogenase [43,44]. *D. hafniense *DCB-2 reduces selenate [Se(VI)] to selenite [Se(IV)] and then to elemental selenium [Se(0)] [6,25]. It is not clear, however, whether selenate reduction is coupled to energy generation in this organism. A homolog for the well-characterized selenate reductase (SER) from *Thauera selenatis *[45,46] was not identified in the DCB-2 genome. However, a putative *dmsABC *operon (Dhaf_1954-1956) that belongs to the same DMSO reductase family of type II molybdoenzymes was significantly induced under selenate-reducing conditions. Interestingly, a putative sulfite reductase α subunit encoded by Dhaf_0252, when produced in *E. coli *BL21-A1 via the expression vector pDEST17, mediated the reduction of selenate but not selenite (data not shown). This gene is part of an eleven-gene dissimilatory sulfite reductase operon (Dsr operon, Dhaf_0251-0261), the products of which catalyze the six-electron reduction of sulfite to sulfide. While sulfite reductase of *Clostridium pasteurianum *and nitrite reductase of *Thauera selenatis *have been implicated in selenite reduction [47,48], selenate reduction by sulfite reductase has not been reported.

Arsenic is readily metabolized by microbes through oxidation/reduction reactions in resistance and respiration processes [49-51]. *D. hafniense *DCB-2 is capable of reducing arsenate [As(V)] to arsenite [As(III)] for respiration [6,25], and the genes for the respiratory arsenate reductase (*arrABC*, Dhaf_1226-1228) are present in its genome. The catalytic subunit, ArrA, contains a molybdenum binding motif that shares a significant homology in amino acid sequence with those of other bacterial respiratory arsenate reductases [51]. Detoxification of arsenic in DCB-2 may be a consequence of arsenic reduction coupled to the arsenite efflux apparatus [49,50]. Three arsenate reductase genes, *arsC*, were identified at different locations (Dhaf_1210, 2269, 2937), and a component for the potential arsenite efflux pump was found as a closely-linked gene (Dhaf_1212).

#### Nitrate reduction

Due to the apparent absence of a Nas assimilatory nitrate reduction system, assimilatory nitrate reduction in DCB-2 appears to be mediated by a five-gene *nap *operon (NapDFBAG, Dhaf-1286-1290) including genes for a periplasmic nitrate reductase NapA (Dhaf_1289) and a 4Fe-4S ferredoxin NapG (Dhaf_1290) [52]. Two copies of an operon encoding NrfAH respiratory nitrite reductase were identified (Dhaf_3630-3631, Dhaf_4234-4235), which catalyzes the one-step conversion of nitrite to ammonia with the generation of energy. NrfA is recognized as a formate-dependent periplasmic cytochrome *c_552 _*and NrfH as a membrane multi-heme cytochrome *c*.

Both *D. hafniense *Y51 and DCB-2 grow well anaerobically with nitrate as the electron acceptor, but only Y51 has the known energy-conserving, respiratory nitrate reduction system (Nar system). The six-gene *nar *operon of Y51 consists of cytoplasmic, respiratory NarGHJI (DSY_0334-0337) nitrate reductase genes and two nitrate/nitrite transporter genes (DSY_0332-0333). The growth of DCB-2 on nitrate (generation time of ~6.5 hrs) may take advantage of the periplasmic Nap system. Nitrite thus formed in the periplasm could be used by the periplasmic, energy-conserving Nrf nitrite reductase without the need to transport nitrate/nitrite across the cytoplasmic membrane. No dedicated nitrate/nitrite transporter gene is found in the DCB-2 genome. The physiological role of a Nap system is often not clear and may vary in different organisms [52]. Another possibility is that an alternative respiratory nitrate reductase may exist in DCB-2. A potential candidate is encoded by Dhaf_0550, which annotated in IMG as nitrate reductase (Figure 4) and shows similarity to a nitrate reductase of *Thermosediminibacter oceani *DSM 16646 in the same *Clostridiales *order. The gene encodes a molybdenum-dependent protein of potential cytoplasmic origin and is linked with a gene for a 4Fe-4S protein. They are found adjacent to a formate/nitrite transporter gene which is part of the formyl-tetrahydrofolate synthesis operon (Dhaf_0553-0555). Genes involved in denitrification were also identified: NorBC-type nitric oxide reductase genes (Dhaf_2253-2254) and a nitrous oxide reductase operon, *nosZDFYL *(Dhaf_0209-0214), potentially enabling conversion of NO to N_2 _via N_2_O. The closest protein sequences for NorB and NosZ were found in *Dethiobacter alkaliphilus *AHT (order *Clostridiales*) and *Geobacillus thermodenitrificans *NG80-2 (order *Bacilliales*), respectively. However, no homolog for the NO-forming nitrite reductase gene was identified. A previous attempt to detect N_2_O in the culture was not successful under nitrate-reducing conditions [4], suggesting that DCB-2 lacks the NO-forming nitrite reductase gene.

#### Dehalorespiration

**Figure 5:**
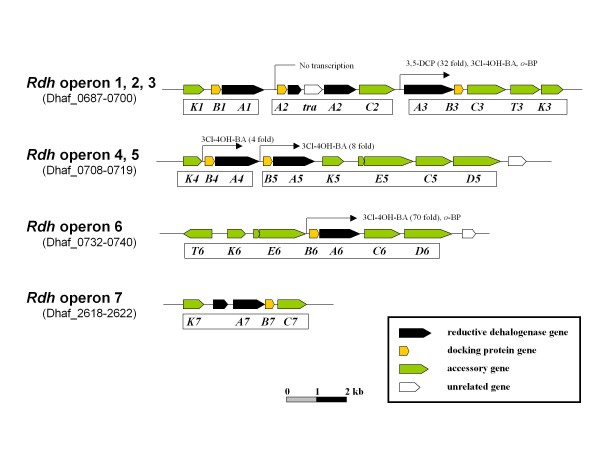
**Physical map of the reductive dehalogenase (*rdh*) operons in *D. hafniense *DCB-2**. The catalytic RDase subunit genes, *rdhA1 *through *rdhA7*, are colored black, and the docking protein genes, *rdhB1 *through *rdhB7*, are colored yellow. Other RDase accessory genes are colored green. Disruptions of *rdhA2 *and *rdhA7 *by an insertion of a transposase gene (*tra*) and by nonsense mutation, respectively, are indicated. The RDase genes, for which transcription was detected by microarrays are indicated with arrows and substrate names with fold induction. All the substrates indicated above the *rdhA2*, *rdhA3*, and *rdhA6 *genes induced their corresponding gene expression in northern hybridization experiments. 3Cl-4OH-BA; 3-chloro-4-hydroxybenzoate, *o*-BP; *ortho*-bromophenol, 3,5-DCP; 3,5-dichlorophenol.

*Desulfitobacterium *and *Dehalococcoides *constitute most of the dehalorespiring bacteria isolates to date. These bacteria can use halogenated compounds such as chlorophenols and chloroethenes as terminal electron acceptors and acquire energy via anaerobic respiration (dehalorespiration). In this process, the halogenated compounds produce halide atoms. *D. hafniense *DCB-2 was isolated using 2,4,6-trichlorophenol (2,4,6-TCP) [4] as its electron acceptor, and also reduces 2,4,5-TCP, 2,4-dichlorophenol (2,4-DCP), 2,5-DCP, 3-chloro-4-hydroxyphenylacetate (3Cl-4OH-PA), tetrachlorohydroquinone (TCHQ), 2,3,5,6-tetrachloro-4-methoxyphenol (TCMP), and pentachlorophenol (PCP) [4,5,53-55]. A slight conversion of tetrachloroethene (PCE) to trichloroethene (TCE) was reported by resting cells pregrown with 3Cl-4OH-PA [53]. In the DCB-2 genome, seven RDase genes were identified (Figure 4) versus two in *D. hafniense *Y51, one of which encodes a PCE RDase (DSY2839, Rdh2 in Figure 1) as it was shown to dechlorinate PCE to *cis*-1,2-dichloroethene via trichloroethene [8,10]. Among the seven DCB-2 RDase genes, *rdhA2 *and *rdhA7 *(Dhaf_0696 and Dhaf_2620) appeared to be non-functional since the genes are interrupted by a transposase gene and nonsense mutation, respectively (Figure 4). BLAST analysis of the five intact genes suggested that four of the genes code for *o*-chlorophenol RDases (*rdhA1, rdhA4, rdhA5, rdhA6*) and *rdhA3 *is highly homologous (66.7% identity in amino acid sequence) to the *pce *gene of Y51 (DSY2839). The operon harboring *rdhA6 *contains a complete gene set for reductive dehalogenation and is similar in gene organization (*cprTKZEBAC*D) to the one in *D. dehalogenans *that is inducible by 3-Cl-4OH-PA [56]. RdhB is an integral membrane protein and acts as a membrane anchor for RDase. RdhC and RdhK belong to the NirI/NosR and CRP-FNR families of transcriptional regulatory proteins. RdhD and RdhE are predicted to be molecular chaperones and RdhT is a homolog to trigger factor folding catalysts. Previously, RDase encoded by *rdhA6 *of DCB-2 was shown to dechlorinate 3-Cl-4OH-PA [57]. We observed, via northern blot analysis, that this gene was also induced in transcription by other halogenated substrates: 3-chloro-4-hydroxybenzoate (3Cl-4OH-BA) and *ortho*-bromophenol (*o*-BP) (summarized in Figure 5). In the same experiment, induction by 3,5-dichlorophenol (3,5-DCP) was observed for *rdhA3 *which was considered to encode a chloroethene RDase. Our cDNA microarray results, obtained from independently prepared samples, were consistent for the high induction of *rdhA6 *by 3Cl-4OH-BA (70-fold) and of *rdhA3 *by 3,5-DCP (32-fold). However, we also observed some inconsistent results between the homology data and the expression data, especially when the level of gene expression was low (e.g. *o*-BP on *rdhA3 *and *rdhA6 *in Figure 5).

### Nitrogen fixation

**Figure 6:**
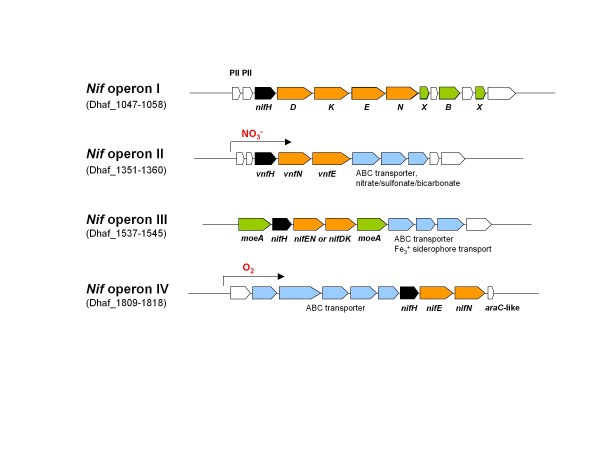
**Physical map of the putative nitrogen fixation (*nif*) operons in *D. hafniense *DCB-2**. The *nifH *homologs are colored black and the homologs for *nifD *or *nifK *are colored orange. Genes involved in MoFe cofactor biosynthesis are colored green; note that *nifK*, *nifE *and *nifN *are also involved in the synthesis of MoFe cofactor. ABC-type transporter genes in the operons are colored blue. The *nif *operon II and IV that were induced in transcription by NO_3_^- ^and O_2_, respectively, are indicated with arrows. PII; nitrogen regulatory protein-encoding gene, *araC*-like; AraC-type transcriptional regulator-encoding gene.

**Figure 7:**
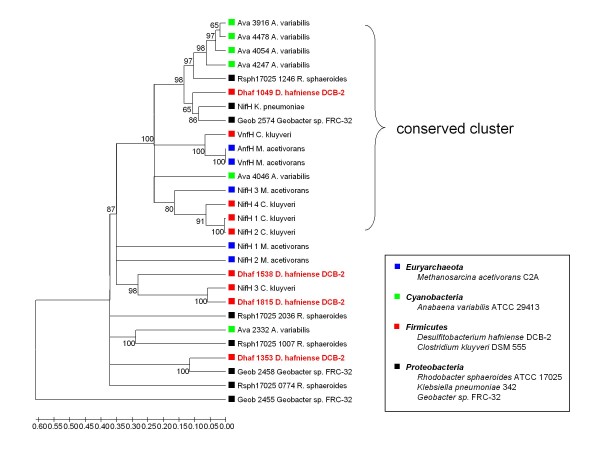
**Phylogenetic tree based on NifH protein sequences**. The tree was derived from 28 NifH protein sequences from six bacterial species and one archaeal species (boxed list), and was constructed using MEGA 4.1 neighbor-joining method with 500 bootstrap replicates. The organisms were chosen from IMG based on their possession of multiple *nifH *gene homologs in their genome except for *Klebsiella pneumoniae *342. The number of *nifH *gene homologs from each species are; five from *Methanosarcina acetivorans *C2A (blue bullets), six from *Anabaena variabilis *ATCC 29413 (green bullets), a total of nine from *Firmicutes *(red bullets); four from *D. hafniense *DCB-2 and five from *Clostridium kluyveri *DSM 555, and a total of eight from *Proteobacteria *(black bullets), including four from *Rhodobacter sphaeroides *ATCC 17025, one from *K. pneumoniae *342, and three from *Geobacter sp*. FRC-32. The tree shows that the NifH encoded by Dhaf_1049 belongs to a more conserved NifH cluster and is distant from other NifH homologs of *D. hafniense *DCB-2.

After noting multiple genes for nitrogenase in the *D. hafniense *DCB-2 genome, we tested the strain for its ability to grow on N_2 _in a medium free of fixed nitrogen (Table 2). The strain readily grew under these conditions and formed cell aggregates tightly bound to the inner surface of a culture bottle. No growth was detected when argon gas instead of N_2 _was used. N_2 _fixation in bacteria is primarily catalyzed by the molybdenum-dependent nitrogenase (Mo-nitrogenase) which is composed of a MoFe nitrogenase complex, NifDK, and a nitrogenase Fe protein, NifH. Four putative *nif *operons were identified in the DCB-2 genome with different sets of associated genes, (Nif operon I-IV, Figure 6) (Dhaf_1047-1059, Dhaf_1350-1360, Dhaf_1537-1545, and Dhaf_1810-1818). Phylogenetic analysis of 28 NifH sequences from selected archaeal and bacterial species that contain multiple *nifH *genes in each genome indicated that Dhaf_1049 belongs to the most conserved group which has at least one *nifH *gene from each species (Figure 7). The operon containing Dhaf_1049 (Nif operon I) harbors, in addition to *nifDK*, genes required for MoFe cofactor biosynthesis and two upstream genes for nitrogen regulatory protein PII, an arrangement similarly found in methanogenic Archaea [58]. Other *nifH *genes of *D. hafniense *DCB-2 (Dhaf_1815 and Dhaf_1353), are distantly related to each other but have close orthologs in *Clostridium kluyveri *DSM 555 and *Geobacter sp*. FRC-32, respectively. We observed that the *nifH *gene and other components of the Nif operon IV including a gene encoding an AraC-type transcriptional regulator (Dhaf_1818) were highly upregulated when cells were exposed to oxygen, suggesting that the operon plays a role in cellular defensive/adaptation mechanisms under oxidative stresses. NifK and NifD encoded by Dhaf_1354-1355 of Nif operon II contain VnfN- and VnfE-like domains that are components of vanadium nitrogenases (V-nitrogenase) of *Azotobacter vinelandii *and *Anabaena variabilis *[59,60]. These proteins may serve as scaffolding proteins for FeV-cofactor synthesis. V-nitrogenases enable cells to fix N_2 _in the presence of vanadium and in the absence of molybdenum. We observed that *D. hafniense *DCB-2 could also fix N_2 _when grown with vanadium in Mo-free medium, a result we also saw in three other dehalorespiring organisms; *D. chlororespirans*, *D. frappieri *PCP-1, and *D. frappieri *DP7 (data not shown). Thus, Nif operon II is implicated in V-dependent N_2 _fixation in *D. hafniense *DCB-2. Microarray studies using different anaerobic respiration conditions indicated that all the *nif *operons in DCB-2 were expressed even when NH_4_^+ ^was used as a major N source. In addition, the Nif operon II which contains a complete set of ABC-type nitrate/sulfonate/bicarbonate transporter genes, a feature unique among bacterial *nif *operons, was highly expressed under nitrate-respiring conditions, making it difficult to predict the primary function of the operon other than its potential role in V-dependent N_2 _fixation.

**Table 2:** Culture conditions of *D. hafniense *DCB-2

Experiments	Basal medium	Carbon/e^- ^donor	e^- ^acceptor/substrate added	Headspace gas	Comments
Pyruvate fermentation	DCB1*, vitamins	Pyruvate, 20 mM		N_2_, 95% CO_2_, 5%	Reference culture for microarray

Fe(III) reduction	CBF**, vitamins	Lactate, 20 mM	Ferric citrate, 50 mM or Ferric oxide, 50 mM	N_2_, 95% CO_2_, 5%	Ferric citrate for microarray Ferric oxide for growth study only

Se(VI) reduction	DCB1, vitamins	Pyruvate, 20 mM	Sodium selenate, 1 mM	N_2_, 95% CO_2_, 5%	For microarray

U(VI) reduction	DCB1, vitamins	Pyruvate, 20 mM	Uranyl acetate, 0.5 mM	N_2_, 95% CO_2_, 5%	For microarray

As(V) reduction	DCB1, vitamins	Pyruvate, 20 mM	Sodium arsenate, 1 mM	N_2_, 95% CO_2_, 5%	For growth study only

Nitrate reduction	CBF, vitamins	Lactate, 20 mM	Potassium nitrate, 10 mM	N_2_, 95% CO_2_, 5%	For microarray

DMSO/TMAO reduction	DCB1, vitamins	Lactate, 20 mM	DMSO, 5 mM or TMAO, 5 mM	N_2_, 95% CO_2_, 5%	For growth study only

3-Cl-4-OH-BA dechlorination	DCB1, vitamins	Pyruvate, 20 mM or Lactate, 20 mM	3-chloro-4-hydroxybenzoate 1 mM or 50 μM for growth	N_2_, 95% CO_2_, 5%	Pyruvate for microarray & northern blot Lactate for growth study

3,5-DCP dechlorination	DCB1, vitamins	Pyruvate, 20 mM or Lactate, 20 mM	3,5-dichlorophenol 1 mM or 50 μM for growth	N_2_, 95% CO_2_, 5%	Pyruvate for microarray & northern blot Lactate for growth study

*o*-BP debromination	DCB1, vitamins	Pyruvate, 20 mM or Lactate, 20 mM	*ortho*-bromophenol 1 mM or 50 μM for growth	N_2_, 95% CO_2_, 5%	Pyruvate for microarray & northern blot Lactate for growth study

Oxygen effect	DCB1, vitamins	Pyruvate, 20 mM		N_2_, 95% CO_2_, 5%	Exposure to air for 3 hours after fermentative cell growth

N_2 _fixation	DCB1, vitamins	Pyruvate, 20 mM		N_2_, 95% CO_2_, 5%	NH_4_^+ ^omitted from DCB1 Gas replenished every 12 h

CO_2 _fixation	DCB1, vitamins			CO, CO_2_ N_2_, H_2_	Details in Figure 3

DCB1*, modified DCB1 medium [61]

CBF**, modified CBF medium [32]

### Oxidative stresses

Although classified as an obligatory anaerobe, *D. hafniense *DCB-2 can tolerate considerable oxygen in liquid culture and can resume its anaerobic growth after 24 hours' exposure to oxygen [4]. Most *Clostridium *species can accept microoxic conditions and are considered to possess systems to metabolize oxygen as well as to scavenge reactive oxygen species (ROS)[62-64]. NoxA, a H_2_O-forming NADH oxidase, has been implicated in oxygen consumption in *Clostridium aminovalericum *[64]. Our total genome microarray study revealed that among four *noxA *homologous genes identified in the DCB-2 genome, a gene encoded by Dhaf_1505, which also showed the lowest E-value of 1e-43, was significantly upregulated upon oxygen exposure (~5 fold). Cytochrome *bd *quinol oxidase (CydA, B), a respiratory cytochrome oxidase unusual for strict anaerobes, was reported to catalyze reduction of low levels of oxygen in the strict anaerobe, *Moorella thermoacetica *[65]. A complete *cyd *operon (*cydA*, *B*, *C*, *D*) was also identified in DCB-2 (Dhaf_1310-1313). However, the operon was not induced under the microoxic conditions that we tested. Under the same conditions, Dhaf_2096 encoding a putative bifunctional catalase/peroxidase was highly upregulated (~12 fold) and the expression of heme catalase-encoding Dhaf_1029 was also considerably induced (~3 fold). No significant induction was observed for three other catalase-encoding genes (Dhaf_1329, Dhaf_1481, and Dhaf_1646) and two Fe/Mn-type superoxide dismutase genes (SOD genes; Dhaf_1236 and Dhaf_2597), although a gel-based cDNA detection study indicated that the Dhaf_1236 SOD gene was expressed constitutively. Other oxygen responsive genes include those for thioredoxin (Dhaf_1227 and Dhaf_3584), thioredoxin reductase (Dhaf_0850), and rubrerythrin (Dhaf_4567). These results suggest that *D. hafniense *DCB-2 is equipped with and can operate defensive machinery against oxygen, which includes ROS scavenging, oxygen metabolism, and other oxygen-responsive reductive activities.

### Sporulation and germination

**Figure 8:**
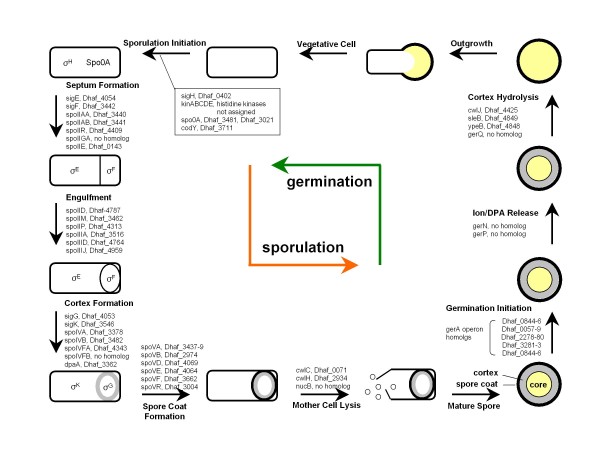
**Putative diagram of sporulation and germination events in *D. hafniense *DCB-2**. The proposed genes are based on known developmental and genetic processes of sporulation and germination in *Bacillus *and *Clostridium *species. A brief description for each developmental stage and the genes encoding stage-specific enzymes or structural proteins are depicted. Compartment-specific sigma factors are also indicated. Gene homologs in *D. hafniense *DCB-2 were identified by using BLASTP with cutoff values of 1e-2 (E-value) and 30% identity in amino acid sequence.

Of the 12 *Desulfitobacterium *strains that have been examined, seven strains including *D. hafniense *DCB-2 were observed to sporulate [1]. Sporulation of *Clostridium *and *Bacillus *involves a cascade gene expression triggered by stage- and compartment-specific sigma factors [66,67]. The genes for the key σ factors (σ^H^, σ^F^, σ^E^, σ^G^, and σ^K^) and the master regulator SpoOA were identified in the genome of DCB-2, and homologs for most of the sporulation genes were identified. Although less conserved, the earliest sporulation genes of sensory histidine kinases could not be positively assigned among 59 histidine kinase genes in the genome (Figure 8). A gene homolog for SpoIIGA, a pro-σ^E ^processing protease, was not identified in either *D. hafniense *DCB-2 or Y51 strains, nor in four other spore-formers of *Peptococcaceae *listed in IMG. However, a homolog for *spoIIR *was identified in all six strains, the product of which could interact with SpoIIGA for the processing of pro-σ^E ^into active σ^E^, a sigma factor responsible for the expression of ~250 genes in the mother cell of *Bacillus subtilis *[68]. Both genes are also present in *Clostridium *spore-formers. Notable *Bacillus *sporulation genes that are missing in *D. hafniense *DCB-2 as well as in *Clostridium *are the genes encoding SpoIVFB, a pro-σ^K ^processing enzyme, SpoIVFA, an inhibitor of SpoIVFB, and NucB, a sporulation-specific extracellular nuclease (Figure 8). This suggests that although sporulation in *Bacillus *and *D. hafniense *DCB-2 have much in common, there are differences in the regulatory mechanism or in the enzyme system for the initiation of sporulation stages.

Germination of spores occurs in response to nutrients (or germinants) which are often single amino acids, sugars or purine nucleosides, and is initiated by binding of germinants to receptors located in the spore's inner membrane [69,70]. In *Bacillus subtilis*, these receptors are encoded by the homologous tricistronic *gerA, gerB *and *gerK *operons [70]. Five such operons were identified in the genome of *D. hafniense *DCB-2 (Figure 8) including an octacistronic operon (Dhaf_0057-64) which encodes additional genes for Orn/Lys/Arg decarboxylase, DNA polymerase III δ' subunit, polymerase suppressor protein, and corrin/porphyrin methyltransferase, suggesting that the operon is used not only for the synthesis of a germinant receptor but for other metabolic activities in relation to sporulation/germination. Upon the binding of receptors to germinants, release of cations and dipicolinic acid (DPA) occurs through hypothetical membrane channels. Potential candidates for such ion/DPA channels were reported as a Na^+^/H^+^-K^+ ^antiporter, GerN of *B. cereus *and GerP proteins of *B. cereus *and *B. subtilis *which are also required for proper assembly of the spore coat [71,72]. No homolog for such genes was identified in *D. hafniense *DCB-2. Specific degradation of the spore's peptidoglycan cortex is mediated by two enzymes, CwlJ and SleB, which require muramic-δ-lactam in peptidoglycan for their action [73,74]. Homologous genes encoding CwlJ and SleB were identified in the genome of *D. hafniense *DCB-2 along with a gene coding for a membrane protein YpeB which is required for SleB insertion into the spore [74,75]. Despite progress in the study of spore germination, little is known about the function of the receptors, signal transduction, and the mechanism of spore-coat breakdown [69,70]. The germination system of *D. hafniense *DCB-2, which lacks some important gene homologs, may provide clues for understanding the missing links in other well-studied systems.

### Biofilm formation

*D. hafniense *DCB-2 was showed to form biofilm in PCP-acclimated bioreactors [55,76] and could also form biofilm on bead matrices under pyruvate fermentative conditions, and even more rapidly under Fe(III)-reducing conditions [25]. Under the identical Fe(III)-reducing conditions but with no added beads, cells expressed genes for type IV pilus biosynthesis (Dhaf_3547-3556) and genes involved in the gluconeogenesis pathway including the fructose-1,6-bisphosphatase gene (Dhaf_4837). Development of microbial biofilm encompasses attachment, microcolony formation, biofilm maturation and dispersion, a series of processes mediated by flagellae, type IV pili, DNA, and exopolysaccharides [77,78]. An increased production of type IV pili and exopolysaccharides would appear to contribute to faster establishment of biofilm under the Fe(III)-respiring conditions.

### Microcompartments

A variety of bacteria utilize ethanolamine, a compound readily available from the degradation of cell membranes, as a source of carbon and/or nitrogen [79]. This process, which occurs within proteinaceous organelles referred to as microcompartments or metabolosomes, involves cleaving ethanolamine into acetaldehyde and ammonia, and a subsequent conversion of acetaldehyde into acetyl-CoA [80]. In *Salmonella typhimurium*, 17 genes involved in the ethanolamine utilization constitute a *eut *operon [80]. All these genes were also identified in the genome of *D. hafniense *DCB-2 but were scattered among four operons (Dhaf_ 0363-0355, Dhaf_4859-4865, Dhaf_4890-4903, and Dhaf_4904-4908). Two genes (*eutBC*) encoding ethanolamine ammonia lyase which converts ethanolamine to acetaldehyde and ammonia were present in one operon (Dhaf_4859-4865), and the *eutE *gene encoding acetaldehyde dehydrogenase which forms acetyl-CoA was found as copies in the other three operons. In addition, five structural genes of microcompartments, *eutS, L, K, M*, and *N *were present separately throughout the four operons, implicating that a concomitant induction of these operons would be required for this structure to function. However, as seen in *Klebsiella pneumoniae *and *Pseudomonas fluorescens*, short operons which contain *eutBC *but not the microcompartment structural genes still function without the benefit of the structure in concentrating acetaldehyde or protecting the cell from its toxic effects [81,82]. In *Enterobacteriaceae *and *Firmicutes*, a full array of *eut *operon (long operon) is generally found [82]. We observed that the two operons designated as Dhaf_4890-4903 and Dhaf_4904-4908 were separated only by 816 nucleotides, and the corresponding region of the *Desulfotomaculum reducens *MI-1 genome (Dred_3264-3286) contained a single contiguous operon of 23 genes, suggesting that an insertion mutation may have occurred in *D. hafniense *DCB-2 in the region between Dhaf_4903 and Dhaf_4904. Finally, the presence of a gene encoding formate C-acetyltransferase within the Dhaf_4904-4908 operon suggests that the *eut *operons of DCB-2 could be used for the synthesis of pyruvate from ethanolamine via acetyl-CoA formation.

### Secretion and transport systems

Although major components for the general secretion (Sec) pathway and the twin-arginine translocation (Tat) pathway are present in *D. hafniense *DCB-2, they differ from those of Gram-negative bacteria [83]. The Sec translocase, a protein pore in the cytoplasmic membrane, which translocates secreted proteins in an unfolded state, appeared to consist of SecY/SecE in this organism (Dhaf_0442/Dhaf_0404) and in other members of *Clostridiales*, whereas a heterotrimer of SecY/SecE/SecG was identified in *E. coli *[84]. In addition, no gene encoding SecB chaperone which guides the secreted proteins to the translocase by binding to an ATP-hydrolyzing SecA (Dhaf_4747) was identified. However, a possible alternative route for guiding the secreted proteins to the translocase, which is mediated by a signal recognition protein (Dhaf_3761) and its receptor (FtsY, encoded by Dhaf_3767), was present. The Tat secretion system is an exporter for folded proteins, often with a redox cofactor already bound, and consists of three membrane proteins, TatA/TatB/TatC in *E. coli *[85]. As in most Gram-positive bacteria, genes encoding only two Tat subunits, a target protein-recognizing TatC protein (Dhaf_3363) and a pore-forming TatA protein, were identified in the DCB-2 genome, with four TatA encoding genes located at different loci (Dhaf_0231, Dhaf_2560, Dhaf_3345, Dhaf_3363).

A total of 733 genes (approximately 14.5% of total CDS) involved in the transport systems of DCB-2, were identified in Transporter Classification of IMG. Among them, 311 encoded proteins belonged to the ATP-Binding Cassette (ABC) superfamily which includes transporters for anions, cations, amino acids, peptides, sugars, polyamines, metal ions, and antibiotics. The genome also encodes ubiquitous secondary active transporters, 47 of which belonged to the Major Facilitator Superfamily (MFS), nine to the RND efflux transporter family, six to the MATE efflux transporter family, and three to the APC superfamily. Seven annotated monocation/proton antiporters and twelve symporters were identified. The presence of multi-copy transporters such as ten sodium/sulfate symporters, eight ABC-type cobalamin/Fe(III)-siderophores transport systems, three *dctPQM *TRAP dicarboxylate transporters, three Fe(II) transporters, and four L-lactate permeases suggests the importance of their substrates in cellular metabolism.

## Conclusions

The genomic analysis of *D. hafniense *DCB-2 described in this paper suggests that the strain is highly self-sufficient in various aspects of metabolism and adaptation. *D. hafniense *Y51 and DCB-2 contain the largest number of molybdopterin oxidoreductase genes known, which suggests that they may impart to these organisms their anaerobic respiration and reduction versatilities. Only a few genes among the 53 Mo-oxidoreductase genes in DCB-2 were identified with a predictable function. Potential electron acceptors used by these enzymes could include, among others, metal ions. Unlike the Gram-negative metal reducers such as *S. oneidensis *MR-1- and *G. sulfurreducens*, in which multi-heme cytochrome *c *proteins were shown to reduce metals, *D. hafniense *DCB-2 contains a very limited number of cytochrome *c *genes. This fact, along with its rich pool of Mo-oxidoreductases, would make this strain a convenient model system for the study of metal reduction in Gram-positive bacteria. Our transcriptomic studies have identified candidate genes for the reduction of Fe(III), Se(VI), and U(VI), suggesting targets for mutant analysis to delineate function. The presence of 19 fumarate reductase paralogs, presumably functioning as dehydrogenase, oxidase, or reductase of unidentified substrates, could also enrich the cell's repertoire of reductive capacities. In addition, *D. hafniense *DCB-2 is likely to possess enzymes or enzyme systems that are novel, as seen in the genetic components for dissimilatory nitrate reduction and nitrogen fixation. The cell's ability to respire nitrate, in the absence of the conventional Nar system, could lead to the elucidation of additional function of the Nap nitrate reductase or to the identification of an alternative system for respiratory nitrate reduction. Similarly, the presence of three additional *nifHDK *homologs, all associated with transporter genes, and their different induction patterns indicate that these operons may have functions other than conventional nitrogen fixation.

Many lines of evidence support the ability of *D. hafniense *DCB-2 to cope with changes of growth conditions and environmental stresses. These include the possession of genes for 59 two-component signal transduction systems, 41 methyl-accepting chemotaxis proteins, 43 RNA polymerase sigma factors, about 730 transporter proteins, and more than 300 transcriptional regulators. Also, motility generated by flagella, endospore formation and germination, tolerance to oxygen, ability to fix CO_2_, and biofilm formation should provide flexible options for *D. hafniense *DCB-2 under stressful conditions. These qualities would make the strain an attractive bioremediation agent in anaerobic environments that are contaminated with nitrate, metal ions, or halogenated compounds.

## Methods

### Culture conditions and genomic DNA extraction

*D. hafniense *DCB-2 cells were grown fermentatively under strict anaerobic conditions on 20 mM pyruvate in a modified DCB-1 medium supplemented with Wolin vitamins [61]. Cultures were incubated at 37°C without shaking under the headspace gas mixture of 95% N_2 _and 5% CO_2_. Cells in mid-logarithmic phase were harvested, and the genomic DNA was isolated according to the procedure of Marmur [86]. Integrity of the genomic DNA and the absence of extrachromosomal DNA elements were confirmed by pulsed field gel electrophoresis (PFGE) and agarose gel electrophoresis.

Culture conditions for the growth and transcription studies are summarized in Table 2. Cell growth under different metal-reducing conditions was monitored by HPLC for consumption of substrates, by optical density that had been previously correlated with the colony forming units and, in the case of some metals, by color change of the culture [25]. Halogenated compounds were added to the fermentatively growing cells (OD_600 _of 0.1), and the cells were allowed to grow for 6 h before harvest for microarray and northern blot analyses. Cells exposed to oxygen were prepared by exposing fermentatively growing cells (OD_600 _of 0.1) to filtered air for 3 h with shaking (60 rpm). Autotrophic cell growth was obtained in a carbon fixation medium which is composed of a modified DCB-1 medium, Wolin vitamins, and different gas mixtures as indicated in Table 2 and Figure 3b. The autotrophic cell growth was examined by cell counts after four transfers to a fresh carbon fixation medium with a growth period of 14 days per transfer. For the biofilm study, cells were grown by fermentation and Fe(III)-respiration (Table 2). Two bead types, activated carbon-coated DuPont beads (3-5 mm diameter) and rough-surfaced silica glass Siran™ beads (2-3 mm diameter) were filled in serum vials. The beads were laid 2.5 cm deep with 1 cm cover of medium, and the medium was refreshed every 2.5 days without disturbing. Biomass and cell size were estimated qualitatively by using light microscopy and scanning electron microscopy from retrieved bead samples.

### Microarray and northern hybridization

Culture conditions for the production of cDNA used on the microarrays are described above and in Table 2. Construction of glass slide arrays and the probe design were performed by the Institute for Environmental Genomics (IEG) at the University of Oklahoma. A total of 4,667 probes covering most of *D. hafniense *DCB-2 genes were spotted in duplicate on a slide, including probes for positive and negative controls. Procedures for RNA extraction, cDNA synthesis and labeling, microarray hybridization and analysis were described by Harzman [25].

Northern hybridization was performed using the DIG DNA Labeling and Detection kit (Roche Applied Science, IN, USA). The RNeasy Midi kit (Qiagen, CA, USA) was used for RNA extraction. Total RNA was isolated from *D. hafniense *DCB-2 grown with 3-chloro-4-hydroxybenzoate, 3,5-dichlorophenol or *ortho*-bromophenol. Samples of 20 μg of RNA were loaded in triplicates on a 1% agarose gel containing 2.2 M formaldehyde. After electrophoresis, the RNA was transferred to a nylon membrane (Hybond-N, GE Healthcare Biosciences, NJ, USA) and each replicate on the membrane was hybridized with the DIG-labeled probes that were designed specifically for targeting the *rdhA2*, *rdhA3*, or *rdhA6 *genes. Hybridization was performed for 16 h at 42°C and positive fragments were detected by chemiluminescence as described in the manufacturer's manual. The microarray data is deposited at GEO-NCBI with the accession numbers GSE33988 and GPL14935 for the raw data and platform, respectively.

### Genome sequencing and annotation

The genome of *D. hafniense *DCB-2 was sequenced by the Joint Genome Institute (JGI). All general aspects of library construction and sequencing performed at the Joint Genome Institute are described at http://www.jgi.doe.gov/. Genome drafts were annotated by the automated pipeline of the Oak Ridge National Laboratory's Computational Genomics Group, and the completed genome sequence of *D. hafniense *DCB-2 has been annotated and curated by the Integrated Microbial Genomes (IMG, http://img.jgi.doe.gov/cgi-bin/w/main.cgi) [87].

### Comparative analysis

Comparative analysis of the microbial genomes and their individual genes were performed with analysis tools and sequence data available at IMG. Topology predictions for signal peptides, transmembrane proteins, and twin-arginine (Tat) signal peptides were performed by using SignalP 3.0 Server (http://www.cbs.dtu.dk/services/SignalP/), TMHMM Server v. 2.0 (http://www.cbs.dtu.dk/services/TMHMM/), and TatP 1.0 Server (http://www.cbs.dtu.dk/services/TatP/), respectively. Alignment of the two *D. hafniense *genomes was performed by using Mauve v 2.3.1 [88] with a view of 24 LCBs (locally collinear blocks) and their GC profiles were obtained by using the GC-Profile program (http://tubic.tju.edu.cn/GC-Profile/), [88,89]. Much of information on metabolic pathways, enzyme reactions, and chemicals were reassured with reference to MetaCyc [90].

### Phylogenetic analysis

Phylogenetic trees of selected proteins were constructed using MEGA 4.1 [91] based on the alignments generated by CLUSTALW algorithm and the neighbor-joining method with 500 bootstrap replications.

### Nucleotide sequence accession number

The sequence data of *D. hafniense *DCB-2 can be accessed using GenBank accession number CP001336.

## Authors' contributions

All authors contributed in the organization and design of experiments as well as data interpretation and manuscript preparation. SHK and JMT wrote the paper. SHK carried out the majority of the genomic analysis. SHK and TLM did the genome comparisons. SHK performed the northern analyses. CH and SHK performed the electron acceptor growth and microarray studies. JKD carried out the early growth, dehalogenase expression and N_2_-fixation studies. CH performed the biofilm studies. RH performed the study of selenate reduction by sulfite reductase. JMT, TLM and JBB conceived of the project, obtained the funding and shaped the experimental design. JMT, TLM and JBB provided laboratory equipment, materials and supervision for the work. All authors read and approved the final version of the manuscript.
